# Anticancer restrictive diets and the risk of psychological distress: Review and perspectives

**DOI:** 10.1002/cam4.7329

**Published:** 2024-07-05

**Authors:** Valentina Da Prat, Gabriella Pravettoni, Amanda Casirati, Chiara Marzorati, Paolo Pedrazzoli, Riccardo Caccialanza

**Affiliations:** ^1^ Clinical Nutrition and Dietetics Unit Fondazione IRCCS Policlinico San Matteo Pavia Italy; ^2^ Applied Research Division for Cognitive and Psychological Science Istituto Europeo di Oncologia IRCCS Milan Italy; ^3^ Department of Oncology and Hemato‐Oncology University of Milan Milan Italy; ^4^ Department of Oncology Fondazione IRCCS Policlinico San Matteo Pavia Italy; ^5^ Department of Internal Medicine and Medical Therapy University of Pavia Pavia Italy

**Keywords:** fast‐mimicking diet, ketogenic diet, nutrition, psychological distress

## Abstract

**Introduction:**

The most studied anticancer restrictive diets include fasting, fasting‐mimicking diets (FMDs) and ketogenic diets (KDs). Besides the current lack of established clinical benefit and the significant risk of malnutrition and micronutrient deficiencies, dietary restrictions in cancer patients might have relevant psychological effects.

**Materials and Methods:**

We reviewed the randomized and non‐randomized controlled clinical trials (CCTs) reporting data on the psychological impact of fasting, FMDs and KDs in cancer patients. We excluded trials on restrictive diets performed for weight reduction in obese or overweight patients, studies on dietary restrictions lasting less than 24 h, and studies on fasting related to cultural or religious beliefs.

**Results:**

Three CCTs on fasting or FMDs and eight CCTs on KDs in cancer patients were included. In terms of diet‐related distress, emotional, social, and family well‐being, none of these studies showed a detrimental impact of fasting, FMDs and KDs. However, clinical trials specifically assessing the psychological aspects in the long term are lacking.

**Conclusions and Perspectives:**

In the absence of a conclusive evidence on the clinical benefits of restrictive diets, which carry significant risks especially if unsupervised, further studies are needed to clarify their psychological impact in cancer patients. Multidisciplinary approaches including psychological evaluations should be used to ameliorate patient selection for clinical trials, identify early distress symptoms, and increase patient compliance to dietary recommendations.

## 1 INTRODUCTION

### 1.1 Restrictive dietary interventions for cancer treatment

In the last decades, several dietary approaches have been proposed with the aim of supporting standard anticancer treatment through the reduction of adverse effects, the improvement of quality of life (QoL), and possibly the enhancement of treatment response. In this setting, restrictive nutritional plans are based on the reduction of certain type of foods and/or on the reduction of caloric load.

If the good sense suggests that a healthy diet (e.g., limitation of junk food, excessive sugar intake, and alcohol) would be beneficial or at least harmless for cancer patients, the risk/benefit ratio of restrictive diets is still debated. Theoretically, restrictive diets may enhance cancer cell death by taking advantage of the consequences of energy metabolism reprogramming, which was included in the hallmarks of cancer sustaining cell proliferation and growth.^1^ In particular, cancer cells preferentially produce energy through anaerobic fermentation of glucose, in contrast to healthy cells, which mainly rely on mitochondrial oxidative phosphorylation, as initially described by Warburg.^2^ Therefore, lowering glucose availability through restriction of caloric and carbohydrate load may hinder cancer cell growth and improve the response to anticancer treatment, as suggested by preclinical studies.^3^, ^4^ In addition, restrictive diets may affect molecular pathways regulating cell proliferation, such as insulin/IGF‐1 or HIF‐1α signaling.^5^, ^6^ Moreover, microbiota modulation induced by restrictive diets may improve the tolerance to anticancer treatment and lead to favorable metabolic and immunologic modifications.^7^

In the last decades, the most studied nutritional approaches in Oncology have been fasting‐based approaches (i.e., fasting and fasting‐mimicking diets [FMDs]) and ketogenic diets (KDs).

Fasting consists of complete food abstinence and ad libitum water intake for a period from a few hours to a few days. Because most patients have difficulties in tolerating fasting for multiple days, FMDs have been developed to enable patients to eat while achieving effects similar to fasting, thanks to very low calorie and low protein nutritional plans.^3^ In humans, cycles of FMDs are usually performed as 3–4 days of a vegan or product‐based diet every month. FMDs have been tested in phase I–II clinical trials in different cancer types combined with standard anticancer treatment, showing the reduction of tumor‐related metabolic and inflammatory factors.^8^ In some cases, an improvement in tumor response compared with standard therapies alone was observed.^9^

KDs include normocaloric and low‐calorie diets based on the increase of dietary fat (up to 70%–80% of total calories) and substantial restriction of carbohydrate intake. KDs are maintained for a period of several weeks to months. They have been associated with overall good safety and tolerability in preclinical and clinical studies, with some reports of enhanced antitumor effects of chemo‐ and radiotherapy.^10^

However, despite the promising results of restrictive diets in the experimental cancer models, studies in humans are not conclusive, in particular due to the lack of assessment of relevant clinical and prognostic endpoints. Therefore, given the very low grade of evidence and the risk for sarcopenia and malnutrition in cancer patients, fasting‐based approaches and KDs are not recommended in the current guidelines for cancer treatment.^11^, ^12^, ^13^

### 1.2 Patients' reasons for undergoing anticancer restrictive diets and potential psychological benefits

Besides the setting of clinical trials, many patients follow restrictive diets that are self‐administered and based on nutritional advice found on the Internet and social media. In fact, the preliminary evidence on the potential benefits of FMDs and KDs and the consequent associated “marketing rumors” have induced many patients to follow low calorie and/or low carbohydrate diets without medical supervision. The limited availability of Clinical Nutrition services in most countries has likely contributed to the increased number of patients following unsupervised nutritional programs, as well as the reluctance of Oncologists to discuss nutritional issues with their patients.^14^

The actual prevalence of cancer patients on restrictive diets is unknown, but is probably high. The NutriNet‐Santé study on more than 2700 cancer survivors found that 6% of patients had fasted in the attempt to improve cancer prognosis. Compared to patients who did not fast, it was less likely that fasting patients received nutritional information from health care professionals. Moreover, 17.4% of cancer survivors thought that the practice of fasting could have a positive impact on cancer prognosis and/or risk of recurrence, despite the absence of conclusive evidence.^15^

In 2019, a French study investigated the motivations to fast among a population of women with breast cancer.^16^ In this setting, patients' primary reason to fast was the attempt to lower the negative side effects of chemotherapy (CT), which were described as a major source of stress. Moreover, fasting was reported as a coping strategy to feel a greater sense of control over anticancer treatment, through active participation and involvement in the healing process. Recent studies on anxiety in patients with breast cancer confirmed a significant association between problem‐focused coping and lower anxiety levels^17^; moreover, a positive association between active problem confrontation and QoL was demonstrated.^18^ Furthermore, studies on KDs in cancer patients showed an improvement in patients' mental wellbeing despite progressive disease status, possibly due to the participation of patients in nutritional treatment as a mean to actively improve their clinical conditions.^19^

Cognitive appraisal and emotional arousal (i.e., emotion's intensity level) play a pivotal role in shaping specific behaviors in cancer care. Indeed, cancer patients often feel powerless and overwhelmed by their uncertain clinical pathway, thus leading to the need of regaining sense of control and creating stability and predictability in their daily routines.^20^ Adopting a restrictive dietary pattern may help them in better managing emotions and gaining a sense of mastery over their bodies and lives. The stringent rules of these diets may also offer an illusionary roadmap, providing structure and order amidst uncertainty^21^; this condition, together with the initial success experienced by individuals following restrictive diets, such as perceived health improvements, can lead to positive reinforcement.^22^ This reinforcement may strengthen the connection between restrictive eating and improved emotional well‐being, creating a cycle of continued adherence.

### 1.3 The hidden risks of restrictive diets

From a clinical perspective, restrictive diets can be potentially harmful at different levels, especially if not supervised by Clinical Nutrition specialists.

Cancer carries a well‐recognized malnutrition risk related both to the tumor itself and to anticancer treatments, through the effects of increased inflammation, cytokine‐induced systemic symptoms limiting food intake (e.g., anorexia, nausea), and potential direct impact on the gastrointestinal tract leading to mechanical or functional impairment (e.g., obstruction, malabsorption).^23^ The prevalence of malnutrition in cancer patients is particularly high, ranging from 20% to 70% depending on the stage, location and clinical setting.^24^ Poor nutritional status is associated with worse outcomes in terms of CT tolerance, rate of post‐surgical complications, length of hospitalization, and survival.^25^ In particular, approximately 10%–20% of patients die as a result of malnutrition rather than cancer.^26^ In the hospital setting, malnourished patients are twice as likely as well‐nourished patients to receive antibiotics for an infection, have longer lengths of stay, higher rates of hospital readmission, significantly worse surgical outcomes, and higher mortality rates, in addition to increased healthcare costs.^27^, ^28^

Restrictive diets can contribute to weight loss, thereby promoting the development of malnutrition, and prolonged avoidance of certain types of foods, such as dairy or animal products, may cause vitamin or mineral deficiencies, if micronutrients are not adequately supplemented. Consequently, restrictive diets carry an inherent risk of worsening rather than improving the prognosis of cancer patients; in extreme cases, unmonitored restrictive diets can even lead to death.

Beyond the physical consequences, restrictive diets may be associated with detrimental psychological effects. Nutritional deficiencies, weight loss, and reduced energy levels due to extreme or unsupported restrictions can significantly affect patients' psychological well‐being; proper nutrition is vital for physical and mental health, and deficiencies can exacerbate emotional issues.^29^ The focus on food intake can increase anxiety about food choices and worries on what to eat, becoming a constant source of stress and rumination. In this vein, scientific literature reports ruminative thinking as a relevant process in the eating disorder psychopathology and/or in the exacerbation of depressive symptoms.^30^ Additionally, a restrictive regimen can affect cancer patients' ability to enjoy food and social interactions.^31^ Dealing with restrictive diets may strictly limit social situations: others may not fully understand dietary needs, thus enhancing feelings of loneliness and stigmatization.^32^

The sense of detachment from their social support networks may greatly impact on patients' mental well‐being and enhance emotional distress: sharing meals with relatives may be experienced as a frustrating experience.^16^ Finally, a recent review on KD for cancer treatment suggested that the incapacity to follow a restrictive diet may be felt as discouraging and demoralizing by patients, especially when the adherence to dietary interventions can be checked by doctors through specific analyses (e.g., ketone bodies dosage in urine and blood which is usually performed for KD monitoring).^33^ Compared to pharmacologic interventions, clinical trials assessing dietary regimens may carry a greater risk of affecting patients' self‐esteem, as discontinuing the diet may be perceived as a matter of lack of willpower and self‐control, with an adverse effect on the overall QoL and mental health of cancer patients.

Therefore, even though nutritional therapy has lower rates of adverse effects when compared to standard anticancer treatment,^34^ the potential psychological risks of restrictive diets should not be underestimated. Unfortunately, psychological symptoms may be difficult to evaluate, since they cannot be properly measured in the animal model and are only seldom assessed in human studies.

## 2 MATERIALS AND METHODS

We reviewed the clinical trials reporting data on the psychological impact of restrictive diets in the literature. In particular, the studies were selected according to PICOS (Participants, Intervention, Comparison and Outcome, and Study design) principles. We included studies on patients diagnosed with any kind of cancer. We decided to focus on fasting, FMDs and KDs, compared with standard/normocaloric diets. We only considered studies that evaluated the psychological impact of restrictive diets, including emotional and social aspects, and distress symptoms. We included randomized and non‐randomized controlled clinical trials (CCTs).

We combined the terms “fast‐mimicking” OR “fasting” OR “ketogenic” to identify the diet component and “cancer” OR “oncolog*” to identify the disease component. The PubMed, Web of Science, and Scopus databases were used as the search platforms (last updated on July 19, 2023); reference lists of the selected articles were searched to identify additional studies.

We excluded trials on restrictive diets performed for weight reduction in obese or overweight cancer patients, studies on dietary restrictions lasting less than 24 h (e.g., pre‐surgical or time‐restricted fasting), studies on fasting related to cultural or religious beliefs (e.g., Ramadan fasting), and studies which lacked a control group.

In most cases, psychological aspects were not specifically evaluated, but were included in QoL questionnaires as questions on emotional, family and social well‐being, and sleep quality, as detailed in Table S1. Other tools specifically assessing psychological aspects in the included trials are described in Table S2.

## 3 RESULTS

### 3.1 Fasting and fasting‐mimicking diets

Three CCTs reported data on the psychological impact of fasting or FMDs in cancer patients (Table 1). In 2018, Bauersfeld and colleagues evaluated the effects of a FMD in patients with breast and ovarian cancer. In this pilot trial, QoL was assessed according to Functional Assessment of Chronic Illness Therapy—Fatigue (FACIT‐F) measurement system, which includes the domains of social/family and emotional well‐being. The FMD consisted of a 60‐h diet of juice, tea and broth before and after CT administration, for three consecutive CT cycles, followed by a normocaloric diet for the successive 3 cycles. Patients in the control arm followed a normocaloric diet for the first 3 cycles of CT, followed by the FMD for the last 3 cycles. FMD was associated with a global improvement in QoL compared to controls; detailed data on individual psychological and emotional aspects were not provided.^35^ Lugtenberg and colleagues investigated the role of a product‐based FMD performed for 3 days prior to CT administration compared to the standard diet in patients with breast cancer. The psychological modifications during FMD were specifically assessed through the Psychological Distress Thermometer (DT) and the Brief Illness Perception Questionnaire (BIPQ), besides being included in the emotional and social domains of the European Organization for Research and Treatment of Cancer (EORTC) QLQ‐C30 questionnaire for QoL measurement. No significant differences between groups were detected neither during diet restriction nor at 6‐month follow‐up; at the exploratory per‐protocol (PP) analysis, patients in the FMD group displayed a significant improvement in the emotional and social fields of QoL questionnaires, in insomnia and in feelings of understanding the illness.^36^, ^37^ Riedinger and colleagues investigated the effects of fasting compared to standard diet in patients with gynecologic malignancies; a QoL evaluation through the National Comprehensive Cancer Network/Functional Assessment of Cancer Therapy Ovarian Cancer Symptom Index (NCCN/FACT) FOSI‐18 questionnaire was performed, including emotional symptoms, general well‐being and sleep quality assessment. No differences in these specific domains were detected, but fasting patients significantly improved global QoL scores from baseline to CT cycles compared to patients following standard diets.^38^

**TABLE 1:** Controlled clinical trials evaluating the psychological impact of fasting and fasting‐mimicking diets (FMDs) in cancer patients.

Reference	Study type, number of patients included in the analysis (experimental/control arm) disease type (therapy)	Intervention	Psychological conditions among exclusion criteria	Psychological effects being evaluated (timepoints)	Results
Bauersfeld et al.,^35^	RCT, *n* = 34 (18/16) Breast and ovarian cancer (CT)	FMD (tea, vegetable juice and broth; 350 kcal) 36 h before and 24 h after CT for cycle 1–3, followed by normocaloric diet for cycle 4–6 vs. vice‐versa (cross‐over)	History of eating disorder; psychosis	‐ Psychological aspects included in FACIT‐F QoL questionnaire (after each CT cycle)	Improvement in global QoL during FMD compared to normocaloric diet (*p* = 0.013) if FMD performed in the first CT cycles (specific data on psychological aspects not reported)
Lugtenberg et al.,^36^ based on de Groot et al.,^37^	RCT, *n* = 112 (54/58) Breast cancer (CT)	FMD (Xentigen™ = plant‐based low amino‐acid substitution diet: soups, broth, liquids and tea; Day 1: 1200 kcal; Days 2–4: 200 kcal) for 3 days prior to and during CT, for 6–8 cycles vs. regular diet	Non‐specific (“medical or psychological condition which would not permit to complete the study or sign meaningful informed consent”)	‐ Distress thermometer (DT); ‐ Brief Illness Perception Questionnaire (BIPQ); ‐ Psychological aspects included in EORTC QLQ‐C30 QoL questionnaire (halfway therapy + end of therapy + at 6‐month follow‐up)	At the ITT analysis, no differences between groups, except for less concerns about disease course in the FMD group (*p* < 0.05). At the exploratory PP analysis, FMD group compared to regular diet had better emotional (*p* = 0.01) and social (*p* = 0.039) functioning scores, improved insomnia (*p* = 0.048) and improved feelings of understanding the illness (*p* = 0.044); no differences in DT; no differences between groups over time
Riedinger et al.,^38^	RCT, *n* = 20 (10/10) Ovarian, uterine, cervical cancer (CT)	Fasting (water, coffee, tea) 24 h before and after CT, for 6 cycles vs. normocaloric diet	No	‐ Psychological aspects included in NCCN FACT FOSI‐18 QoL questionnaire (after each CT cycle)	No differences between groups in mean scores, single psychological aspects, and insomnia; greater improvement in global QoL over time in fasting group compared to controls (*p* = 0.015).

*Note*: Questionnaires and psychological evaluation tools are detailed in Tables S1 and S2.

Abbreviations: BIPQ, Brief Illness Perception Questionnaire; CT, chemotherapy; DT, distress thermometer; EORTC, European Organization for Research and Treatment of Cancer; FACIT‐F, Functional Assessment of Chronic Illness Therapy; FMD, fasting‐mimicking diet; ITT, intention to treat; NCCN, National Comprehensive Cancer Network; QoL, quality of life; RCT, randomized clinical trial.

Overall, only one of these three studies mentioned eating disorders as an exclusion criteria for enrolment.^35^

### 3.2 Ketogenic diets

Eight CCTs reported data on the psychological impact of KDs on cancer patients (Table 2).

**TABLE 2:** Controlled clinical trials evaluating the psychological impact of ketogenic diets on cancer patients.

Reference	Study type, number of patients included in the analysis (experimental arm/control arm) disease type (therapy)	Intervention	Psychological conditions among exclusion criteria	Psychological effects being evaluated (timepoints)	Results
Augustus et al.,^19^	RCT, *n* = 36 (16/20) Any cancer type (CT)	KD (10% C, 15% p, 75% F) for 16 weeks vs. standard diet	Preexisting psychosocial conditions such as moderate/severe depression/anxiety	‐ Psychological aspects included in EORTC QLQ‐C30 QoL questionnaire ‐ Depressive symptoms according to Patient Health Questionnaire (PHQ‐9) (2–6‐12 weeks + end of intervention)	Substantial improvement in depressive symptoms and global QoL scores in the KD group over time (*p* < 0.05); compared to controls, improvement in depressive symptoms (*p* < 0.0001) and global QoL score (*p* = 0.0001)
Cohen et al.,^39^	RCT, *n* = 45 (25/20) Ovarian and endometrial cancer (CT or no therapy)	KD (5% C, 25% p, 70% F) for 12 weeks vs. normocaloric diet according to American Cancer Society guidelines	Major depressive or psychiatric disorder; anti‐psychotic agents, monoamine oxidase inhibitors	‐ Psychological aspects included in the 12‐Item Short Form Survey (SF‐12); ‐ Food craving according to Food Craving Inventory (FCI) (end of intervention)	No differences between groups in mental health status; KD group reported less frequent cravings compared to baseline (*p* = 0.0004) and, with respect to salty foods, to control group (*p* = 0.03)
Kämmerer et al.,^40^	Non‐randomized controlled trial, *n* = 152 (29/92/31) Breast cancer (rehabilitation)	KD (2%–4% C, 16%–18% p, 80%–85% F) for 20 weeks vs. low carbohydrate diet (20–30% C, 20–30% *p*, 40–50% F) for 20 weeks vs. standard diet	Non‐specific (“Dementia or other clinically relevant alterations of the mental status which could impair the ability of the patient to apply to the diet or understand the informed consent”)	‐ Psychological aspects included in EORTC QLQ‐C30 QoL questionnaire (end of intervention)	Compared to baseline, improved emotional functioning in KD (*p* = 0.006) and low carbohydrate (<0.0001) groups, improved social functioning in low carbohydrate group (*p* = 0.002), improved insomnia in KD group (*p* = 0.01). Highest emotional functioning and lowest insomnia scores in KD group (*p* value not shown)
Khodabakhshi et al.,^41^	RCT, *n* = 80 (40/40) Breast cancer (CT)	KD (6% C, 19% *p*, 75% F) for 90 days vs. standard diet	No	‐ Psychological aspects included in EORTC QLQ‐C30 QoL questionnaire (6 weeks + end of intervention)	No differences in insomnia, emotional and social functioning compared to baseline and to control group (which improved in social functioning compared to baseline)
Klement et al.,^42^	Non‐randomized controlled trial, *n* = 59 (29/30) Breast cancer (RT)	KD (C < 50 g/day, F 75%–80%) during RT (several weeks) vs. standard diet according to the German Nutrition Society	Psychological disorders	‐ Psychological aspects included in EORTC QLQ‐C30 QoL questionnaire (halfway therapy + end of therapy)	At the end of therapy, improvement in insomnia (*p* = 0.0072), emotional (*p* = 0.00017) and social functioning (*p* = 0.0098) compared to baseline in the KD group; no differences compared to control group
Klement et al.,^43^	Non‐randomized controlled trial, *n* = 41 (18/23) Rectal cancer (CT‐RT)	KD (C < 50 g/day, F 75%–80%) during CT‐RT (several weeks) vs. standard diet according to the German Nutrition Society	Psychological disorders	‐ Psychological aspects included in EORTC QLQ‐C30 QoL questionnaire (halfway therapy + end of therapy)	At the end of therapy, improvement in social functioning (*p* = 0.0089) compared to baseline in the KD group; no differences compared to control group
Voss et al.,^44^	RCT, *n* = 50 (25/25) Glioblastoma/gliosarcoma (RT)	3‐day of hypocaloric KD (C < 50 g/day), then 3‐days fasting, then 3‐days of of hypocaloric KD (as above) before and during 5/10‐day RT vs. standard diet according to the German Nutrition Society	Non‐specific (“dementia or other clinically relevant alterations of the mental status which could impair the ability of the patient to apply to the diet or understand the informed consent”)	‐ Psychological aspects included in EORTC QLQ‐C30 QoL questionnaire ‐ Psychological symptoms according to Symptom Check List‐90 (SCL‐90) (Day 6 + 12)	No significant differences (data on SCL‐90 and QoL questionnaires not shown)
Zorn et al.,^45^	Non‐randomized controlled trial, *n* = 20 (7/9/1/13) Any cancer type (CT)	Low calorie (400–600 kcal) KD (C 10%, *p* 15%, F 75%) 3 days before and 1 day after CT for 2–3 cycles + standard diet for successive 2–3 cycles vs. vice‐versa (cross‐over) vs. normocaloric KD for 6 days then low calorie KD for 2–3 cycles and standard diet for successive 2–3 cycles vs. vice‐versa (cross‐over)	Eating disorders	‐ Psychological aspects included in EORTC QLQ‐C30 and FACIT‐F QoL questionnaires ‐ Insomnia and depression according to CTCAE grading (after each CT cycle + at 3‐week follow‐up)	No differences between groups in global QoL (specific data on psychological aspects not reported), depression, or insomnia

*Note*: Questionnaires and psychological evaluation tools are detailed in Tables S1 and S2.

Abbreviations: C, carbohydrate; CT, chemotherapy; CTCAE, Common Terminology Criteria for Adverse Events; EORTC, European Organization for Research and Treatment of Cancer; F, fat; KD, ketogenic diet; P, protein; QoL, quality of life; RCT, randomized clinical trial; RT, radiotherapy.

Two trials specifically included psychological reactions among the outcomes, relying on Patient Health Questionnaire 9 (PHQ‐9) for the assessment of depressive symptoms^19^ and on Symptom Check List‐90 (SCL‐90) for psychopathology features.^44^ Compared to baseline, patients in the KD group had a significant improvement in depressive symptoms according to PHQ‐9 during the intervention compared both to baseline and to control group,^19^ while results regarding SCL‐90 were not reported in the ERGO2 trial.^44^

In other trials, psychological aspects were not specifically evaluated, but were included in QoL assessment by EORTC QLQ‐C30 questionnaires or 12‐Item Short Form Survey (SF‐12). In a study by Kämmerer and colleagues, breast cancer patients following a KD or a low carbohydrate diet achieved improvements in emotional functioning (*p* = 0.006 and <0.0001, respectively) during the intervention compared to baseline. In the same study, KD patients showed an improvement in insomnia (*p* = 0.01), while patients on low carbohydrate improved in social functioning (*p* = 0.002) compared to baseline.^40^ Similarly, KD led to an improvement in insomnia, emotional and social functioning compared to baseline in another trial on breast cancer patients undergoing radiotherapy;^42^ in a cohort of rectal cancer patients, KD was associated with better social functioning (*p* = 0.0089) compared to baseline.^43^ Other trials did not find significant differences in the emotional and social domains of QoL or in global QoL with KD,^41^ but none of them suggested a detrimental effect of restrictive diets. When evaluating CT‐related adverse effects, the severity of insomnia and depression were not significantly different among cancer patients following a normocaloric KD, a more restrictive FMD consisting of a low calorie KD, or a standard diet.^45^ Moreover, a study by Cohen and colleagues found a lower prevalence of diet‐related cravings through the Food Craving Inventory (FCI) in patients following a KD compared to patients on standard diet.^39^

Among the eight CCTs, only one study specifically mentioned eating disorders as exclusion criteria.^45^ Two trials excluded patients with psychological disorders,^42^, ^43^ and other two trials reported preexisting psychosocial conditions (such as moderate/severe depression/anxiety), major depressive or psychiatric disorder, and psychiatric therapy as exclusion criteria.^19^, ^36^

## 4 DISCUSSION

In general, small sample sizes, differences in the choice of outcomes, and heterogeneity of patients, cancer treatments, and diseases make it difficult to evaluate the psychological consequences of restrictive diets in the reviewed studies. Moreover, most clinical trials were not designed to assess the impact of dietary interventions in terms of psychological effects. With these limitations, none of the available trials demonstrated an association between restrictive diets and psychological distress, depression, insomnia, emotional, or social/family functioning deterioration during the study period.

However, the psychological impact of restrictive diets may require a longer follow‐up to be fully evaluated, in order to detect late‐appearance symptoms, as food restriction may lead to psychological distress not only during the intervention, but also in the following months/years.

Among the reviewed trials, only two studies provided a follow‐up evaluation at 6 months and 3 weeks after the end of dietary restriction, respectively.^36^, ^45^ The assessment of late psychological effects is particularly relevant for patients with high probability of long‐term survival, such as breast cancer patients, who are the best candidates for restrictive diets due to low prevalence of malnutrition, but may develop diet‐related psychological disturbances after the end of anticancer treatment.

Furthermore, adequately controlled trials are needed to detect the psychological effects of restrictive diets, in order to reduce confounding factors. Studies designed to assess efficacy or physical adverse effects of diets may often include mild food restrictions for patients in the control arm (i.e., controls receive nutritional advice on healthy food or are prescribed normocaloric dietary plans), thus potentially creating a certain degree of diet‐related psychological distress also in the control group. Moreover, dietary counseling is not always provided to patients in control groups, with a possible impact on patients' anxiety. Sometimes patients in control groups have admitted that they practiced food restriction on their own initiative, thus hindering the comparison between groups; for example, in the study by De Groot and colleagues, nearly 8% of patients of the control group decided to fast during one or more cycles of CT.^37^

Finally, dexamethasone administration, which is usually performed to prevent CT‐related side effects, was omitted in patients undergoing restrictive diets in one study due to possible reduction of the diet‐related metabolic and endocrine effects, whereas it was provided in the control group.^36^, ^46^ This difference could have an impact on anxiety, insomnia and other psychological reactions in the short term after CT administration due to the effects of steroids on behavior, thus possibly interfering with the evaluation of the psychological impact of the dietary intervention.^47^

## 5 CONCLUSIONS AND PERSPECTIVES

Restrictive diets not only carry the risk of physical adverse events, such as muscle mass loss and malnutrition, but may also be associated with emotional distress and psychological disturbances in cancer patients.

In the literature, the majority of CCTs did not focus specifically on the psychological impact of restrictive diets. Studies that evaluated the psychological symptoms or the emotional and social/family well‐being in the setting of QoL assessment showed a positive or neutral effect of KDs and FMDs, but clinical trials with longer follow‐up and more adequate control groups are needed for specifically assessing the psychological consequences of restrictive diets.

From a clinical point of view, it is fundamental that nutritional programs are developed by specialized personnel, customized on the single patient, and adequately monitored. In addition, appropriate educational interventions should be implemented to help cancer patients understand the malnutrition risks associated with restrictive diets, and to dispel common myths, such as the possibility of completely depriving cancer cells of their glucose supply.^48^

With respect to the psychological aspects, dietary interventions should be managed with a multidisciplinary approach including a psychological evaluation before, during, and after nutritional treatment. In fact, an accurate selection of patients before prescribing any restrictive diet should be mandatory, in order to appropriately assess the potential clinical benefits, if any, and to exclude individuals with history of an eating disorder or risk factors for developing diet‐related psychological disturbances. During dietary treatment, patients should be monitored in order to identify early psychological symptoms. Compliance could be improved through specialized support aimed at facilitating the active involvement of patients and caregivers, since adherence to dietary restrictions has a tight relationship with the emotional impact of the diet itself. Finally, after the end of any dietary restriction, patients should be psychologically supported in case of fear or anxiety related to the resumption of the usual diet.

It remains implicit that every effort should be made to prevent malnutrition and loss of muscle mass during cancer treatment.

## AUTHOR CONTRIBUTIONS

**Valentina Da Prat:** Conceptualization (equal); methodology (equal); writing – original draft (equal). **Gabriella Pravettoni:** Conceptualization (equal); writing – original draft (equal). **Amanda Casirati:** Writing – review and editing (equal). **Chiara Marzorati:** Writing – review and editing (equal). **Paolo Pedrazzoli:** Supervision (equal); writing – review and editing (equal). **Riccardo Caccialanza:** Conceptualization (equal); supervision (equal); writing – review and editing (equal).

## FUNDING INFORMATION

This work was partially supported by ACC (Bando Ricerca Corrente Reti IRCCS 2021, grant number RCR‐2021‐23671213 and Bando Ricerca Corrente Reti IRCCS 2022, grant number RCR‐2022‐23682293).

## CONFLICT OF INTEREST STATEMENT

The authors declare that they have no known competing financial interests or personal relationships that could have appeared to influence the work reported in this article.

## Supporting information


Table S1.
Table S2.

## Data Availability

Data sharing is not applicable to this article as no new data were created or analyzed in this study.
