# Supplementary material for: Anticancer restrictive diets and the risk of psychological distress: Review and perspectives
Source: Cancer Med. 2024 Jul 5;13(13):e7329. doi: 10.1002/cam4.7329 (PMC11226408; doi:10.1002/cam4.7329)
Supplement: Supplementary file 1 — Table S1. Table S2. [file CAM4-13-e7329-s001.docx]

**Supplementary Table S1***.* Quality of life questionnaires including questions on psychological well-being.

| **Tool** | **Reference** | **Description and psychology-related questions included in the questionnaire** |
| --- | --- | --- |
| EORTC QLQ-C30 questionnaire (Version 3) | Aaronson NK et al, J Natl Cancer Inst (1993) | The EORTC- QLQ-C30 includes 30 items covering five functional scales (physical functioning; role functioning; emotional functioning*; cognitive functioning; social functioning**), nine symptom scales or single items (fatigue; nausea; pain; dyspnea; insomnia***; appetite loss; constipation; diarrhea; financial difficulties), and one global health status scale.  **During the past week: Did you feel tense? Did you worry? Did you feel irritable? Did you feel depressed?*  ***During the past week: Has your physical condition or medical treatment interfered with your family life? Has your physical condition or medical treatment interfered with your social activities?*  ****During the past week: Have you had trouble sleeping?* |
| FACIT-F questionnaire (Version 4) | Webster K et al, Health Qual Life Outcomes (2003) | FACIT-F includes a generic core questionnaire named Functional Assessment of Cancer Therapy-General (FACT-G), evaluating physical, emotional*, social/family** and functional well-being, and an additional questionnaire assessing self-reported fatigue.  **I feel sad; I am satisfied with how I am coping with my illness; I am losing hope in the fight against my illness; I feel nervous; I worry about dying; I worry that my condition will get worse **I feel close to my friends; I get emotional support from my family; I get support from my friends; My family has accepted my illness; I am satisfied with family communication about my illness; I feel close to my partner (or the person who is my main support).* |
| NCCN-FACT FOSI-18 questionnaire (Version 2) | Shaunfield et al, Health Qual Life Outcomes (2019) | The NCCN-FACT FOSI-18 questionnaire belongs to the FACIT© measurement system; it evaluates disease related emotional* symptoms, physical symptoms (including insomnia), treatment side effects, and general function and well-being**.  **I worry that my condition will get worse*  ***I am content with the quality of my life right now* |
| Short Form-12 health survey (SF-12) questionnaire | Ware J et al, Med Care (1996) | SF-12 is used to measure physical and mental health status. Responses to the questionnaire items are scored to produce a physical and a mental* component summary.  **During the past week, have you had any of the following problems with your work or other regular daily activities as a result of any emotional problems (such as feeling depressed or anxious)? Accomplished less than you would like; Didn't do work as carefully as usual.*  *How much of the time during the past week: Have you felt calm and peaceful? Have you felt downhearted and blue?*  *During the past week, how much of the time has your physical health or emotional problems interfered with your social activities (like visiting with friends, relatives, etc.)?* |

**Supplementary Table S2***.* Specific tools evaluating psychological aspects.

| **Tool** | **Reference** | **Description** |
| --- | --- | --- |
| Distress Thermometer (DT) | Tuinman MA et al, Cancer (2008) | Visual analog scale (a thermometer) for evaluation of psychological distress in the past week in patients with cancer, with range 0–10. |
| Brief Illness Perception Questionnaire (BIPQ) | Broadbent E et al, J Psychosom Res (2006) | The BIPQ questionnaire measures eight dimensions of illness perceptions:  - Understanding (how well do you feel you understand your illness)  - Consequences (how much does your illness affect your life)  - Timeline (how long do you think your illness will last)  - Personal control (how much control do you feel you have over your illness)  - Treatment control (how much do you think your treatment can help your illness)  - Identity (how much do you experience symptoms from your illness)  - Concern (how concerned are you about your illness)  - Emotional representation (how much does your illness affect you emotionally) |
| Food Cravings Inventory (FCI) | White MA et al, Obes Res (2002) | The FCI evaluates food cravings in relation to four categories: high-fat foods, sweets, starches, and “fast food fats” (e.g., pizza, hamburgers, and potato chips). The scoring of the questionnaire yields craving scores for each category as well as an overall craving score. |
| Patient Health Questionnaire (PHQ-9) | Manea L et al, Gen Hosp Psychiatry (2015) | The PHQ-9 evaluates the presence of DSM (Diagnostic and Statistical Manual of Mental Disorders) depression diagnostic criteria with other leading major depressive symptoms. |
| Symptom Check List-90 (SCL-90) | Derogatis LR et al. in The use of psychological testing for treatment planning and outcome assessment (1994), Lawrence Erlbaum Associates, Inc. | The SCL-90 is used to assess the following aspects of psychopathology: somatization, obsessive-compulsive behavior, interpersonal sensibility, depression, anxiety, anger-hostility, phobic-anxiety, paranoid ideation, psychoticism. |
